# Solar park promoted microbial nitrogen and phosphorus cycle potentials but reduced soil prokaryotic diversity and network stability in alpine desert ecosystem

**DOI:** 10.3389/fmicb.2022.976335

**Published:** 2022-09-08

**Authors:** Yu Liu, Chengxiang Ding, Derong Su, Tiemei Wang, Tao Wang

**Affiliations:** ^1^College of Grassland, Beijing Forestry University, Beijing, China; ^2^Academy of Animal Husbandry and Veterinary Science, Qinghai University, Xining, China

**Keywords:** solar park, prokaryote and fungi, network stability, microbial function, enzyme

## Abstract

Solar park (SP) is rapidly growing throughout the planet due to the increasing demand for low-carbon energy, which represents a remarkable global land-use change with implications for the hosting ecosystems. Despite dozens of studies estimating the environmental impacts of SP based on local microclimate and vegetation, responses of soil microbial interactions and nutrient cycle potentials remain poorly understood. To bridge this gap, we investigated the diversity, community structure, complexity, and stability of co-occurrence network and soil enzyme activities of soil prokaryotes and fungi in habitats of ambient, the first, and sixth year since solar park establishment. Results revealed different response patterns of prokaryotes and fungi. SP led to significant differences in both prokaryotic and fungal community structures but only reduced prokaryotic alpha diversity significantly. Co-occurrence network analysis revealed a unimodal pattern of prokaryotic network features and more resistance of fungal networks to environmental variations. Microbial nitrogen and phosphorus cycle potentials were higher in SP and their variances were more explained by network features than by diversity and environmental characteristics. Our findings revealed for the first time the significant impacts of SP on soil prokaryotic and fungal stability and functional potentials, which provides a microbial insight for impact evaluation and evidence for the optimization of solar park management to maximize the delivery of ecosystem services from this growing land use.

## Introduction

Demand for clean energy has experienced an exponential increase in the past decade and is expected to increase in the future to contribute to limiting the global temperature rise to 1.5°C (Teske, 2019). Of all the existing renewable energy technologies, solar park has the greatest potential for power generation, which has grown more than five times in the last decade (Yue et al., 2021) and will outpace all other alternative energy sources by 2050 (Osaki, 2019). With such a huge interest in solar energy development, it is crucial to take enough precautions regarding the environmental changes induced by the solar park. The solar park generally consists of large scale of ground-mounted photovoltaic arrays, which could shade habitat, absorb solar radiation, intercept wind, and divert rainfall to the downslope edge. These changes could impose dramatic implications on microclimate, biodiversity, soil nutrients, and diverse ecosystem services (Grodsky and Hernandez, 2020). For instance, reduced mean daily albedo under photovoltaic arrays resulted in lower soil and air temperature (Yang et al., 2017). Declined water evaporation intensity under photovoltaic panels promoted soil water content and water-use efficiency by 328% (Hassanpour Adeh et al., 2018). Lower evaporation, higher soil water content and moderate temperature increased vegetation coverage and aboveground biomass under photovoltaic arrays, which indicated positive effects of SP on the hosted ecosystem (Jiang et al., 2019). However, land-use changes due to large solar facilities establishment can elicit biodiversity loss and thereby reduce ecosystem stability and services (Grodsky and Hernandez, 2020). For instance, solar parks in the desert ecosystem may inhibit native plant species, which could result in a decrease in the historically speciose plant communities underpinning primary productions (Moore et al., 2017). It is worth noting that both the positive and negative effects solar park induced could exert influences on soil microorganisms, nonetheless, how the soil microbial community responds to these perturbations remains poorly understood.

A healthy ecosystem has been defined as *“being stable and sustainable, maintaining its organization and autonomy over time and its resilience to stress”* (Rapport et al., 1998). Environmental perturbations such as climate change can impact the stability and structure of the ecosystem and further affect the ecosystem’s function or services (Kéfi et al., 2019). Finding sensitive and robust indicators in response to environmental perturbations allows humans to better identify, quantify, and anticipate the modifications of ecosystem quality. The biological components are more sensitive to environmental perturbations relative to the abiotic components of the ecosystem (Carignan and Villard, 2002). Within the biological component, microorganisms are one of the most popular bioindicators evaluating the effects of climate changes and anthropogenic perturbations on the ecosystem due to their short generation time and genetic plasticity (Bouchez et al., 2016) and their primary roles in many biogeochemical processes (Singh et al., 2020). Microbial taxonomic diversity and community composition have been the primary bioindicators of land use change (Bouchez et al., 2016), while an increasing number of studies found that the complex interconnections among community members were more sensitive to environmental disturbances (Karimi et al., 2017; Ramirez et al., 2018). In addition, the microbial network could better reveal the changes in the stability of microbiomes, which are influenced by the antagonistic, neutral, and cooperative interactions between the members within these communities (Faust and Raes, 2012). These characteristics indicate that microbial networks could play a complementary role to traditional bioindicators of the impacts of environmental perturbations and ecosystem quality. Previous studies have partially explored the impacts of the solar park on soil archaea and prokaryotic diversity and community composition, while few empirical studies have investigated the changes in complexity and stability of soil microbial co-occurrence network (Chao et al., 2016). It is necessary to include network analysis for a comprehensive assessment of SP effects on soil microbial communities in light of network analysis that has been widely applied in many land use change evaluations and has been proved effective and complementary.

Soil extracellular enzymes make great contributions to soil ecological processes including organic matter decomposition, nutrient cycling, and soil fertility (Acosta-Martínez et al., 2007). Since most of these functions are microbially mediated, soil extracellular enzyme activities (EEAs) can be indicative of microbial function potentials. α-1,4-gulcosidase (αg), β-1,4-glucosidase (βG) and β-xylosidase (βx), β-1,4-N-acetylgucosaminidase (nag), leucine aminopeptidase (lap), and acidic phosphatase (ap) were the most used soil extracellular enzymes related to soil C, N, and P cycle, which could be used as a proxy for soil microbial function potentials (Xiao et al., 2018). It is reasonable to assume that microbial network topological features play an important role in regulating soil EEAs given observed significant associations between network indices and other proxies of microbial function potentials such as the C cycle function gene (Yuan et al., 2021). Despite increasing findings on significant correlations between soil EEAs and microbial community structures as well as environmental changes (Moghimian et al., 2017; Moscatelli et al., 2018), limited studies investigate the impacts of microbial co-occurrence network complexity on this proxy of microbial function potentials.

The solar park will continue to expand for the goal of carbon neutrality. It is necessary to understand the mechanism of SP on a local ecosystem to develop better management practices that mitigate adverse solar park impacts. Nonetheless, previous studies of ecological impacts of SP most concentrated on local microclimate and/or vegetation, ignoring stability of soil microbial interactions network and microbial functional potentials. To bridge this gap, we selected sites of ambient (Y0), the first (Y1), and the sixth year (Y6) since the installation of SP in the alpine desert grassland in Qinghai-Tibet Plateau and investigated the plant and soil physicochemical properties, soil extracellular enzymes activities, and soil microbial matrix. Our study addressed the following questions: (1) Does the solar park alter soil prokaryotic and fungal diversity, community composition, and network complexity and stability; (2) Does the solar park promote or declined microbial function potentials represented by soil EEAs; (3) Does abiotic or biotic factors affect microbial function potentials and which factors regulate them the most.

## Materials and methods

### Site description and soil sampling

**FIGURE 1:**
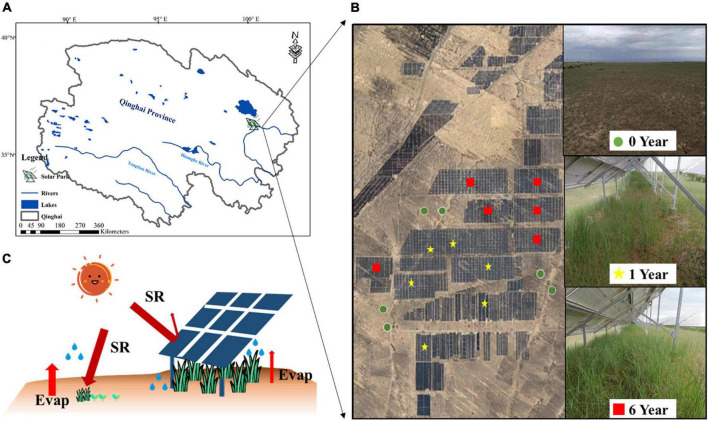
Location of solar park **(A)**, image of sample sites **(B)**, and schematic of the potential effects of photovoltaic panel on microclimate **(C)**.

This research was undertaken at the Huanghe Solar Park, one of the world’s largest solar parks, Longyangxia Dam Solar Park, established in 2013 with a capacity of 850 MW, which is to the southeast of the Qinghai-Tibet Plateau (36°9′47″N, 100°35′14″E) (Figure 1A). The photovoltaic panels are oriented south-facing at an angle of 39°. The photovoltaic panel dimensions are 16.5 m by 9.91 m. The gap between solar panel rows should be around 5.4 m. Before conversion to a solar park, the field site was alpine desert grassland. With an average annual temperature of 4.8–6.0°C, average annual precipitation of 278–523 mm, and annual evaporation of 1,716.7 mm, this region has a semi-arid climate. The soil texture comprises mostly sandy loam. The dominant plant species are *Achnatherum splendens* and *Artemisia frigida*.

To determine the impacts of the solar park on plant-soil properties and soil microbial community, we selected a total of 18 sites of 0 year (Y0), 1 year (Y1), and 6 years (Y6) since SP installation in the alpine desert grassland in Qinghai-Tibet Plateau (Figure 1B). The schematic of photovoltaic panels is shown in Figure 1C. Five randomly distributed soil cores in each site at 0–10 cm depth were collected. Soil cores collected from the same plot were mixed as a composite to minimize the variability caused by soil spatial heterogeneity. Each soil composite was divided into subsamples for separate microbial and chemical analyses. Samples for microbial analysis were saved in sterile bags to avoid contamination and stored in −20°C for DNA extraction, and samples for chemical analysis were put into plastic bags and stored at 4°C. Soil and plant samples of the solar park were sampled under photovoltaic panels. Microclimate, vegetation, soil properties, and soil microorganism metrics of each site were measured.

### Soil physicochemical and plant properties

Soil total organic carbon (SOC), total nitrogen (TN), and total phosphorus (TP) contents were measured with air-dried soils using the K_2_Cr_2_O7–H_2_SO4 titrimetric method, Kjeldahl digestion, and vanadium molybdate yellow colorimetry, respectively. About 2.5 g of fresh soils were extracted with 2 M KCl and filtered to determine soil inorganic nitrogen concentration including nitrate (NO_3_^–^) and ammonium (NH_4_^+^) using the Clever Chem 200+. The microbial biomass carbon (MBC) and nitrogen (MBN) were determined by the chloroform fumigation and extraction method (Davidson et al., 1989). Soil pH was measured with a fresh soil to water ratio of 1:5 using a Sartorius pH meter (PB–10, Sartorius Corporate Administration GmbH, Göttingen, Germany). Soil moisture (SWC) was evaluated by drying the soil at 105°C for 24 h. Soil temperature (ST) was measured at depths of 10 cm using a Hydra Probe sensor (Campbell Scientific, Inc., United States).

A 50 cm × 50 cm quadrat was randomly selected in each plot in mid-August 2019 to measure vegetation biomass (AGB), cover, and diversity. The aboveground vegetation of each plot was oven-dried at 75°C for 48 h to calculate the plant productivity. Vegetation Shannon index (VegS), Vegetation Richness index (VegR), and Vegetation Pielou index (VegP) were measured according to the number of individual species before all plants were clipped.

### DNA extraction and sequence processing

Soil DNA was extracted from 0.5 g frozen soil samples using a FastDNA SPIN Kit for Soil (MP Biomedicals, Santa Ana, CA, United States) according to the instruction manual and was quantified using a NanoDrop 2000 Spectrophotometer (Bio-Rad Laboratories Inc., United States). PCR was carried out using the primer set 515F and 806R for prokaryotic and ITS1F and ITS2 targeting the ITS1 region for fungi. Approximately 250 bp paired-end reads were generated on the Illumina MiSeq platform. The QIIME2 (version 2018.6) was used to analyze the raw sequencing data (Bolyen et al., 2019). The DADA2 plugin in QIIME2 (Callahan et al., 2016) was used to filter out low-quality sequences and chimeras and then generate amplicon sequence variants (ASVs), which were classified using the QIIME2 naive Bayes classifier (Bokulich et al., 2018) trained on 99% Operational Taxonomic Units (OTUs) from the SILVA rRNA database (v 132) (Quast et al., 2012) and UNITE database (Nilsson et al., 2019) for prokaryotic and fungi, respectively.

### Data filtering and alpha and beta diversity analysis

ASVs with very small counts in very few samples are likely due to sequencing errors or low-level contaminations. To ensure a more strict analysis, we only keep ASVs occurring in at least 20% of total samples and containing at least four counts at each sample. All downstream data analysis will be based on filtered data. Shannon and phylogenetic of prokaryotic and fungal alpha diversity indices were estimated. To reveal potential microbial compositional variation across soil samples, PCoA (Principal Coordinate Analysis) was performed based on weighted UniFrac distance and Bray–Curtis distance and Permutational Multivariate Analysis of Variance (PERMANOVA) was used to evaluate significant differences in microbial community structures in a vegan R package (Dixon, 2003).

### Microbial co-occurrences network analyses

The co-occurrence network in most studies was constructed using arbitrary thresholds, and therefore, the constructed networks are subjective rather than objective. To avoid the subjectivity of researchers, a random matrix theory (RMT)-based approach, which can identify a threshold for microbial network construction, was performed to construct the prokaryotic and fungal co-occurrences networks through the Molecular Ecological Network Analyses Pipeline^1^ and were visualized using gephi.^2^ The network analysis is performed according to previous studies by Zhou et al. (2010) and Deng et al. (2012). Topologic features of the network were measured to evaluate the network complexity.

Network modularity (Grilli et al., 2016), network vulnerability (Yuan et al., 2021), and ratio of negative cohesion to positive cohesion (Neg:Pos cohesion) (Herren and Mcmahon, 2017) have been used as the proxy for network stability. Moreover, theoretical studies indicated that natural connectivity with node loss caused by external disturbance could be indicative of network robustness or deletion stability (Dunne et al., 2002). We estimate the network robustness based on the decreasing proportions of natural connectivity and the average degree of the whole network in the case of removing keystone nodes and random nodes (Wu et al., 2021).

Network vulnerability. The vulnerability of a network is indicated by the maximal vulnerability of nodes in the network as follows:


$$\text{max}\,\left(\frac{E-{\text{E}}_{\text{i}}}{\text{E}}\right)$$

where E is the global efficiency and Ei is the global efficiency after removing node i, and d(i,j) is the number of edges in the shortest path between node i and j.


$$\text{E}=\frac{1}{n\,\left(n-1\right)}\,\sum_{\text{i}\neq \text{j}}\frac{1}{d\,\left(i,j\right)}$$

Natural connectivity is calculated based on the following function. λ corresponds to the “average eigenvalue” of the graph adjacency matrix and N is the number of nodes in matrix.


$$\bar{\lambda }=\text{ln}\,\left(\frac{1}{\text{N}}\,\sum_{\text{i}=1}^{\text{N}}{\text{e}}^{{}_{\text{i}}}\right)$$

Network keystone nodes were identified based on (1) hub nodes: Network hubs, connetors, and module hubs classified based on within-module connectivity (Zi) and among-module connectivity (Pi) (Olesen et al., 2006) and (2) Top 10 nodes: nodes with top 10% degree. The R code for network robustness calculation can be found in Supplementary material.

### Microbial function potentials

To estimate the biogeochemical function of microbes, soil extracellular enzymes activity (EEA) involved in C, N, and P turnover were measured, including C-acquisition enzymes, α-1,4-gulcosidase (αg), β-1,4-glucosidase (βG), and β-xylosidase (βx), N-acquisition enzymes, β-1,4-*N*-acetylgucosaminidase (nag), leucine aminopeptidase (lap), and phosphorous-acquisition enzyme, acidic phosphatase (ap) (Jian et al., 2016). In addition, we acquired enzyme metabolic pathways based on Kyoto Encyclopedia of Genes and Genomes orthologs (KOs) and Enzyme Commission numbers (EC numbers) based on 16s sequences with PICRUST2 (Douglas et al., 2020).

### Statistical analysis

To quantify the effects of soil properties, vegetation characteristics, and microclimate on soil microbial diversity, Redundancy analysis (RDA) and Forward Selection with permutation were performed by using the vegan R package. To find the correlations among microbial functional potentials, microbial network features, microbial community structure, and environmental characteristics, Mantel test based on “spearman” with 999 permutations were conducted. To evaluate how the environmental characteristics and microorganisms might have regulated microbial function potentials, VPA was used to discern the contributions of these variables to the overall variations of the microbial function potentials. Canonical Correlation Analysis (CCA) by CANOCO software v4.54 were used to estimate the effects of the environmental factors on microbial communities (MBC, MBN, alpha-diversity of prokaryotes and fungi, etc.).

## Results

### Vegetation and soil physicochemical properties

As shown in Table 1, SP had a significant impact on most environmental factors except soil TP, TK, NO_3_^–^, and pH. The SOC, TN, SWC, MBC, MBN, AGB, and Cover were the highest under the Y6 group and were the lowest under the Y0 group, whereas the Evap and three vegetation diversity indices under Y0 samples were significantly higher.

**TABLE 1:** Environmental factors in Y0, Y1, and Y6 soil samples.

Environmental factors	Y0	Y1	Y6
AGB/g⋅m^–2^	38.83 ± 4.87c	88.84 ± 10.72b	145.34 ± 6.13a
Cover/%	28.83 ± 9.09c	43.83 ± 12.66b	76.83 ± 8.73a
VegR	7.00 ± 0.00a	4.50 ± 0.84b	4.00 ± 0.89b
VegS	1.02 ± 0.11a	0.34 ± 0.25b	0.14 ± 0.12b
VegP	0.93 ± 0.10a	0.33 ± 0.22b	0.16 ± 0.14b
SOC/g⋅kg^–1^	10.83 ± 3.61b	18.02 ± 4.91a	18.01 ± 4.83a
TN/g⋅kg^–1^	0.95 ± 0.09b	1.34 ± 0.17a	1.24 ± 0.12a
**TP/g⋅kg** ^–^ **^1^**	0.49 ± 0.13a	0.56 ± 0.12a	0.57 ± 0.11a
**TK/mg⋅kg** ^–^ **^1^**	18.00 ± 1.79a	18.97 ± 1.80a	20.02 ± 1.94a
NH_4_^+^/mg⋅kg^–1^	4.60 ± 0.54b	6.49 ± 0.96a	6.25 ± 1.34a
**NO_3_^–^/mg⋅kg** ^–^ **^1^**	15.99 ± 1.72a	15.35 ± 5.51a	20.44 ± 12.56a
MBC/mg⋅kg^–1^	9.61 ± 2.51b	20.68 ± 9.00a	24.17 ± 9.90a
MBN/mg⋅kg^–1^	2.91 ± 1.47b	6.55 ± 1.32a	5.68 ± 3.22a
**pH**	8.43 ± 0.23a	8.65 ± 0.22a	8.58 ± 0.41a
SWC/%	6.17 ± 0.09c	8.37 ± 0.22b	11.84 ± 0.15a
ST/°C	19.36 ± 3.85a	14.51 ± 2.70b	12.55 ± 1.65b
Evap/g⋅m^–3^	4.56 ± 1.47a	2.40 ± 0.54b	2.06 ± 1.13b

Environmental factors marked in bold indicate statistically significant differences (P < 0.05) among Y0, Y1, and Y6. AGB indicate plant above-ground biomass; Cover indicate plant cover; VegR, VegS, and VegP indicate plant richness index, plant Shannon diversity index, and plant pielou evenness index, respectively; SOC indicate soil total organic carbon; TN indicate soil total nitrogen; TP indicate soil total phosphorus; NO_3_^–^ indicate soil nitrate nitrogen; NH_4_^+^ indicate soil ammonium nitrogen; MBC and MBN indicate microbial biomass carbon and nitrogen, respectively. The different letters represent the “ambient”, “initial”, and “constant” are corresponding to “Y0”, “Y1”, and “Y6”, respectively.

### Soil microbial diversity and community composition

We characterized 1,531 prokaryotic and 550 fungi species-level ASVs. The majority of the prokaryotic ASVs were classified as Actinobacteria (33.6%), Proteobacteria (23.6%), Acidobacteria (10.7%), followed by Gemmatimonadetes (9.8%) and Thaumarchaeota (9.1%) at the phylum level (Supplementary Figure 1). Phylum Ascomycota (75.8%), Mortierellomycota (16.3%), and Basidiomycota (6.9%) dominated the sample fungi community.

**FIGURE 2:**
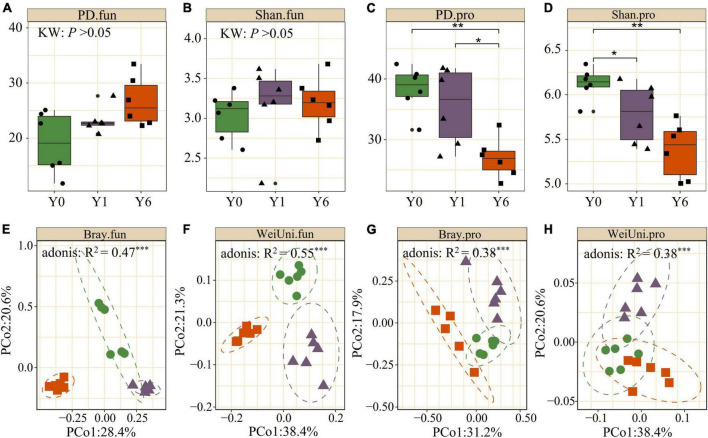
Comparison of alpha and beta diversity across Y0, Y1, and Y6 soil samples. **(A–D)** are phylogenetic index (PD) and Shannon index (Shan) of soil fungal (.fun) and prokaryotic (.pro) communities. **(E–H)** are principle coordinate analysis (PCoA) of soil fungal and prokaryotic communities based on Weighted UniFrac (WeiUni) distance and Bray–Curtis (Bray) distance, respectively. * indicate *p* < 0.05, ** indicate *p* < 0.01, *** indicate *p* < 0.001.

We observed significant differences in α diversity across Y0, Y1, and Y6 in soil prokaryotic communities and Y6 had the lowest Shannon and phylogenetic diversity (*p* <0.05; Kruskal–Wallis). Soil fungi diversity under SP had greater Shannon diversity and PD diversity than the Y0 fungi community, but showed no significant difference, as shown in Figures 2A–D. Both the weighted-UniFrac and Bray–Curtis PCoA showed that prokaryotic and fungi communities from SP soil were clearly separated from the Y0 soil. First, two axes explained 50∼60% total variation for both prokaryotic and fungal communities. Permutational multivariate analysis of variance (Adonis) also identified the significant difference among the three microbial communities. These results indicated the great influence of SP on the belowground microbiota (Figures 2E–H).

### Complexity and stability of soil microbial co-occurrence networks

**FIGURE 3:**
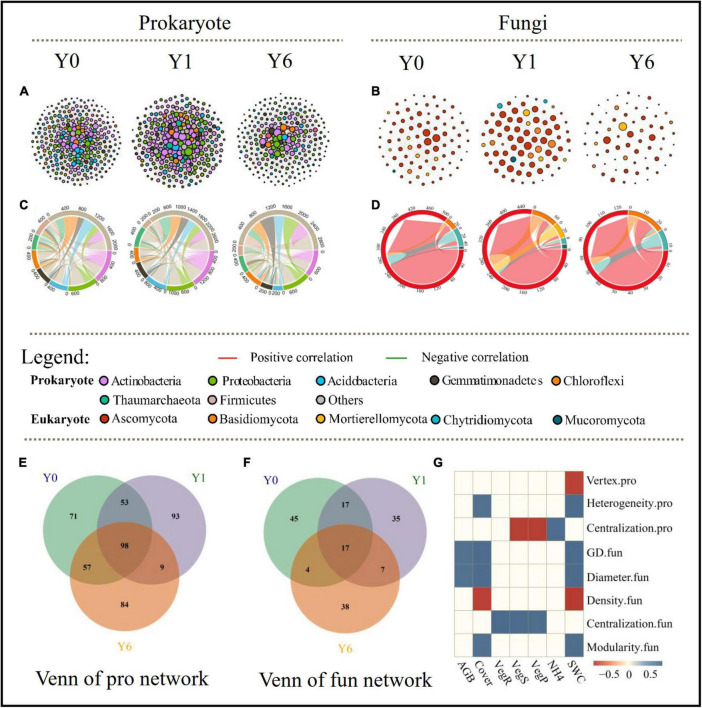
Co-occurrence networks of soil prokaryotic and fungi communities **(A,B)**. Each point represents node (ASV) and each line (link) represents pairwise interaction between nodes, and node colors and size represent phyla and degree, respectively, red and green line indicate the positive and negative correlation between nodes, respectively. The chord diagram shows links among phyla in different network **(C,D)**. The chord color is identical with the nodes in networks. The chord width between two phyla is proportional to the number of associations. **(E,F)** represent venn graphs of nodes in prokaryotic networks and fungal networks, respectively. **(G)** represents the significant correlations between network features and environmental characteristics, network features with suffix “.pro” and “.fun” indicate prokaryotic and fungal networks features, respectively.

As shown in Figure 3 and Table 2, prokaryotic interaction networks with 279, 253, and 248 nodes and fungal interaction networks with 83, 76, and 66 nodes were constructed from Y0, Y1, and Y6 soil samples, respectively. For prokaryotic networks, the Y1 network with the highest average degree (AvgD), average clustering coefficient (AvgCC), and shortest average path distance (GD) were more connecting than Y0 and Y6 networks. For fungal networks, the Y6 had lower links (83) relative to Y0 (296) and Y1 networks (298), and the lowest AvgD and AvgCC and the longest GD, Y0, and Y1 had similar network structures.

**TABLE 2:** Topological properties of prokaryotic and fungi interaction network.

Network properties	Prokaryote			Fungi		
		
	Y0	Y1	Y6	Y0	Y1	Y6
Total nodes	279	253	248	83	76	66
Total links	1329	2053	1122	296	298	83
Average degree (AvgD)	9.53	16.23	9.05	6.67	7.84	2.52
Average clustering coefficient (AvgCC)	0.04	0.08	0.05	0.07	0.12	0.05
Average path distance (GD)	2.74	2.27	2.72	2.51	2.33	4.84
Density (D)	0.03	0.06	0.03	0.08	0.11	0.03
Modularity	0.285	0.284	0.297	0.33	0.29	0.64
Neg:Pos cohesion	0.69	0.68	0.71	0.34	0.38	0.40
NC slope—remove random nodes	−0.09	−0.16	−0.09	−0.05	−0.05	−0.01
AvgD slope—remove random nodes	−0.06	−0.14	−0.07	−0.07	−0.07	−0.03
MDP of NC—remove Top10/%	1.73	1.60	2.33	1.22	1.85	3.27
MDP of NC—remove Hub/%	0.46	0.47	0.56	1.54	1.54	1.02
MDP of AvgD—remove Top10/%	1.26	1.38	1.86	1.94	2.48	3.17
MDP of AvgD—remove Hub/%	0.46	0.48	0.57	1.61	1.57	1.20

NC slope—remove random nodes, slope of the regression of NC with removal of random nodes; MDP of NC—remove Hub, mean declined proportions of natural connectivity since removing hub nodes; MDP of NC—remove Top 10, mean declined proportions of natural connectivity since removing Top 10 nodes.

**FIGURE 4:**
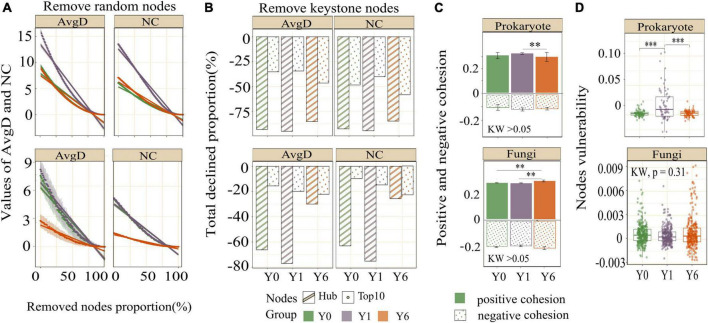
The network stability of three networks. Figures in the first row are the results of prokaryotes and figures in the second row are the results of fungi. Images in panel **(A)** are the AvgD and NC with the removal of random nodes, images in panel **(B)** are the total declined proportions of AvgD and NC since removing all keystone nodes, images in panel **(C)** are the negative and positive cohesion, and images in panel **(D)** are the node vulnerability. *, **, and *** indicate significant differences at 0.05, 0.01, and 0.001 level, respectively.

Network modularity, vulnerability, and ratio of negative cohesion to positive cohesion (Neg:Pos Cohesion) are used most to indicate the robustness or stability of the interaction network. As shown in Table 2 and Figure 4C, both prokaryotic and fungal networks of Y6 had the largest modularity and Neg:Pos Cohesion. The network vulnerability showed a fluctuation in prokaryotic networks but no variation in fungal networks (Figure 4D), indicating higher resistance to disturbances in fungal networks. In addition, we simulated species extinction to estimate network robustness (the resistance to node loss) based on either random node loss or removal of keystone nodes (hub nodes and Top 10 nodes). For prokaryotic networks, both NC and AvgD of Y1 experienced the fastest drop (a bigger absolute value of slope) with the nodes removed randomly increasing (Figure 4A). For fungal networks, Y1 and Y0 showed a similar trend and Y6 showed the lowest slope (Figure 4A). As shown in Figure 4B and Table 2, fungal networks had larger mean declined proportions (MDP) of NC and AvgD but less total declined proportions (TDP) than prokaryotic in general after the removal of keystone nodes. Y6 prokaryotic network had the least TDP of NC and AvgD but had the largest MDP after the removal of hub nodes or Top 10 nodes. Y6 fungal network showed the lowest TDP and MDP of NC and AvgD after hub nodes removal (Figure 4B) but had the highest values after the loss of Top 10 nodes.

### The soil extracellular enzyme activities and microbial functional

**FIGURE 5:**
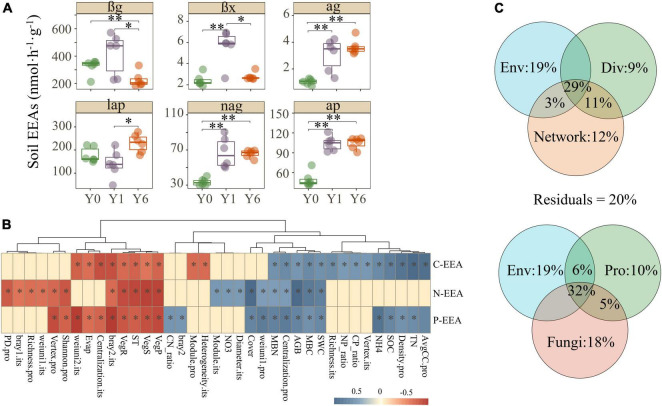
Soil C, N, and P EEAs and their correlations with environmental characteristics and microbial matrix. Images in panel **(A)** are the Soil C, N, and P EEAs, images in panel **(B)** are the correlations among soil EEAs, environmental characteristics, and microbial matrix, and images in panel **(C)** are the variance portioning analysis. Features with suffix “.pro” and “.fun” indicate prokaryotic and fungal features, respectively. *, **, and ** indicate significant differences at 0.05, 0.01, and 0.001 level, respectively.

In general, soil αg, βx, nag, and ap enzyme activities enhanced with the years of solar park establishment, lap decreased in Y1 but achieved the highest value in Y6, and βg revealed a contrary trend with other enzymes which had significant growth in Y1 but dropped to the lowest in Y6 (Figure 5A). These findings indicated a potential promotion of SP on soil EEAs. Mantel analysis was carried out to disentangle the explicit relationships between explained variables and soil C, N, and P enzyme activities. As shown in Figure 5B and Supplementary Figure 4, carbon, nitrogen, and phosphorus EEAs all had significant correlations with environmental characteristics, alpha and beta diversities, and partial network attributes of both prokaryotes and fungi. VPA showed major portions (>80%) of the soil EEAs variations were explained by environmental properties, microbial diversity, and microbial network attributes (Figure 5C). Network attributes contributed most to the variance of soil EEAs, but environmental properties explained the most alone. The diversity and network attributes of fungi explained more variance of soil EEAs than that of prokaryotes (Figure 5C).

## Discussion

Responses of soil microbial matrix to SP were analyzed from different aspects: microbial diversity and community structure, complexity and stability of microbial co-occurrence network, and microbial function potentials.

### Soil microbial diversity and community structure

Soil evaporation under the solar park declined due to the interception of shortwave radiation by the photovoltaic panel (Weinstock and Appelbaum, 2009), which further promoted the soil water content under the solar park. The decrease in prokaryotic diversity under SP possibly stemmed from higher soil moisture, which has been observed in many studies that increased soil water content (Tan et al., 2020) or enhancing precipitation regime significantly reduced soil bacterial diversity (Zhang et al., 2016). The negative correlation between SWC and alpha diversity (Supplementary Figure 2) indicates that parts of soil prokaryotic taxa that survived in the dry ecosystem may not adapt to the moist conditions (Zhao et al., 2018). No significant difference in fungal diversity was found in this study (Figure 2), which may result from their morphological life form: fungi are generally considered more resistant to soil water fluctuation than bacteria (Barnard et al., 2013). A global fungi diversity survey from 15,000 topsoil samples suggested that the crucial factors influencing fungi diversity differed among phylogenetic and functional groups of fungi and that soil pH was typically the most effective edaphic predictor of fungal richness in genera (Tedersoo et al., 2014). We found that solar park influenced the local microclimate most and vegetation but had little effect on soil pH (Table 1). That may also be the reason for no significant difference in fungi diversity.

We found significant impacts of soil water content on the prokaryotic community composition and a significant reduction of soil water evaporation in solar park habitats (Table 1 and Supplementary Figure 5). These results suggested that regulations of SP on the structure of the microbial residents via the mediation of habitat arid index attributed to reduced soil water evaporation and/or absorption of solar radiation in desert grassland. Prior studies have found the significant impacts of the solar park on soil archaea and prokaryotic community composition, and this study found significant separation among fungi communities after solar park installment (Figure 2), indicating that SP could mediate not only prokaryotic communities but fungal communities. Given the intimated correlations between environmental properties and fungal taxa, the variations of fungal community structures could mainly be because the changed environment in SP habitats mediated the relative abundance of fungal taxa, such as phyla Ascomycota and Mortierellomycota (Supplementary Figure 1). Mantel test showed that soil water content, plant diversity, and biomass had great correlations with soil fungal community structure (Supplementary Table 2), which is consistent with the results of precipitation control experiments (Xiao et al., 2020).

### Complexity and stability of microbial co-occurrence networks

The distinct responses of prokaryotic and fungal communities to solar parks in water-limited areas provide fundamental information for our understanding of microbial network robustness under the background of global climate change. In the present study, we used a series of network topological parameters and diverse indicators related to network robustness to reveal the microbial interactions (if correlations between taxa are treated as putative interactions). Our results supported the proposed hypothesis that changed environmental factors induced by solar parks had a time effect on the complexity and robustness of soil microbial co-occurrence networks. This hypothesis was based, in part, on previous findings that altered soil water contents and years could strongly impact the complexity and robustness of co-occurrence relationships among microbes (Yuan et al., 2021; Li et al., 2022). Indeed, the overall topological properties of prokaryotic networks changed with SP years and showed a unimodal pattern rather than a linear model. The results are similar to prior reports that robustness of co-occurrence relationships had a dramatic shift in the second year since watering and then network robustness tended to recover to the previous trends observed in the first year (Shi et al., 2020). These results suggest that the prokaryotic network may have pulse responses in the case of emerging soil water content increase, but they will return to the initial state with the adaptation to the soil water conditions. This trend has also been observed in the responses of microbial community composition and CO_2_ emissions to SWC variation (Barnard et al., 2013). Higher SWC promoted dominant species abundance and made them outcompete disadvantaged species, which weakened the robustness of the network (Shi et al., 2020). With the extension of the precipitation period, SWC would turn into a stress factor for the soil microbes and then result in more competing relationships among microbes, which enhanced the network robustness in the fourth year. In this study, we tend to assume changing interactions among microbes rather than microbial community composition altered network robustness (Ratzke et al., 2020). Several biogeographic studies and field experiments have pointed out that, as with larger organisms, microbes are dispersal-limited (Hanson et al., 2012). Outstanding dispersal abilities are capable of promoting species interactions (Lemes et al., 2022). Increased soil water availability in SP habitats enhanced the dispersal ability of prokaryotic species and provided them more opportunities to interact with each other, which may explain higher network connectivity. High connectivity resulted in a decrease in the robustness of the Y1 network (Pietro et al., 2018). The recovery of the Y6 network may be attributed to microbial adaptation to the habitat. Adaptive behavior of species in response to environmental changes (Strona and Lafferty, 2016) can often form new links. Food-web structures have been observed adaptive networks (Nuwagaba et al., 2015), which promote stability (Nuwagaba et al., 2017). Several studies have reported that microbes are capable of adapting to shifting environments after long periods of exposure (Tan et al., 2022). Therefore, we assumed that microbial adaptation to higher SWC modified the network interactions and then enhanced the network robustness after 6 years of exposure to SP. These assumptions were evidenced by similar network nodes and changing interactive relationships among microbes across three networks (Figure 3E).

Different models of fungi networks detected that network complexity and robustness had few shifts a year since SP installment, indicating a stronger network resistance of fungi than the prokaryotic network to environmental changes (de Vries et al., 2018). This result is consistent with the prior assumption that prokaryotes are typically more sensitive than fungi to water variation (Manzoni et al., 2012). Y6 showed a more robust fungal network relative to Y1 and Y0, as evidenced by less connectivity and larger modularity, and an increased ratio of negative cohesion to positive cohesion. In addition, relatively flat drops of NC and AvgD in the Y6 network compared with Y1 and Y0 since the loss of random nodes may indicate increasing robustness of the fungal network to random species extinction. Recent experimental works have suggested that keystone taxa exert direct impacts on soil fungal network stability (Shen et al., 2022). The differences in declined proportions of NC and AvgD between removal of hub nodes and removal of Top 10 nodes could be attributed to the large differences in network features among the three networks, especially modularity. The modularity of the Y6 fungal network was two-fold that of the other two networks. The high modularity ensures perturbations experienced by members are harder to propagate through the entire community, and thus the loss of hub nodes has fewer effects on network robustness (Grilli et al., 2016). The greater loss of NC and AvgD after removal of Top 10 nodes in Y6 may result from the higher proportions of the node degree of Top 10 nodes to all nodes in Y6 (24.5%) relative to Y1 (15.7%) and Y0 (19.5%), given that the algorithm estimating network robustness is correlated with nodes degree. The results suggest that the impacts of keystone nodes on microbial co-occurrence network robustness depend on the definition of keystone nodes and network complexity.

### Biogeochemical cycle and microbial function

In this study, we combined changes in soil extracellular enzyme activities to illustrate the impact of the solar park on microbial function potentials and link them with environmental factors, microbial matrix including alpha diversity, community structures, and network topological features to disentangle the dominant driving factors.

Soil extracellular enzymes play a crucial role in soil biological processes such as the degradation and mineralization of organic carbon, and the nutrient cycle including C, N, and P (Baldrian, 2009). Moreover, the rapid responses of soil EEAs to external disturbances make them a sensitive indicator of soil quality evaluation (Asadishad et al., 2018; Cao et al., 2020). Our results revealed that SP increased most soil EEAs except βg related to carbon, nitrogen, and phosphorous cycle (Figure 5A). The decreased βg enzyme activities may attribute to reduced microbial taxa abundance for observed significantly lower EC 3.2.1.2 in the Y6 habitat based on results of functional prediction of the KEGG pathway (Supplementary Figure 4). The increasing soil EEAs in SP habitats could attribute to their positive correlations with soil nutrients conditions including SWC, SOC, TN, MBC, and MBN (Figure 5B), which was in agreement with prior studies that improved nutrients condition could promote soil enzyme activities (Xiao et al., 2019). Meanwhile, we found diversity and community structure of both soil prokaryotes and fungi also affected soil EEAs (Figure 5B), indicating soil microbial communities play a critical role in mediating soil EEAs (Roux et al., 2013; Xu et al., 2021). Many researchers are increasingly aware that microbial communities mediate soil enzyme activities in the means of regulating taxa abundance related to soil enzyme synthesis and activity in the disturbed environment (Burns et al., 2013). This study found significant correlations between microbial taxa abundance at the phyla level and soil EEAs (Supplementary Figure 6), such as the significant positive correlation between phyla Ascomycota and βg enzyme activity. Ascomycota comprises multiple fungal taxa that can produce a set of enzymes that decompose cellulose to glucose (Karkehabadi et al., 2014; Méndez-Líter et al., 2020). These results suggested that solar parks could regulate soil EEAs via changing relative abundances of prokaryotes and fungal species related to soil extracellular enzyme production (Krishnan et al., 2011).

An intriguing question is whether the network topological features affect microbial functional potentials. We used various analyses to address it. First, the Mantel test showed that the network features linked tightly with C, N, and P cycle EEAs, particularly C cycle EEAs (Figure 5C). Recent studies in diverse ecosystems have found similar trends that the topological characteristics of co-occurrence networks could be associated with the observed microbial functional potentials or ecological functions. An experiment conducted on the grassland ecosystem reported more than half of carbon (C) degradation genes had significant correlations with soil microbial network indices after long-term warming (Yuan et al., 2021). An investigation of anaerobic digestion system showed that microbial network properties such as GD and AvgCC correlated with high-hydrolysis efficiency (51.8–80.5%) and methanogenesis efficiency (51.6–77.1%) (Guo et al., 2022). VPA also revealed that topological features totally explained 55% variances of soil EEAs, more than environmental parameters (51%) and microbial diversity (49%) (Figure 5B). This result indicates that the microbial co-occurrence network could regulate microbial functions and it is possible that the changes in network complexity serve as early indications of microbial functions. Notably, these results indicate only the intimate correlations between microbial co-occurrence network features and microbial functions, and more and deep investigations should be carried out to disentangle the mechanism.

These differential responses of soil EEAs to SP may indicate that the regulation of environmental changes on soil microbial function potentials has two ways: one is the bottom-up regulation caused by shifts of vegetation composition and soil environment, and the other one is the top-down controls exerted by the producers of soil enzymes, soil microbes, through changing diversity, and community composition and interactive relationships.

## Conclusion

In conclusion, our results indicate that solar park could alter soil prokaryotic and fungal diversity, community structure, co-occurrence network, and function potential. Soil water content was the major factor regulating prokaryotic community. Fungal communities were more intensively affected by soil nutrients and vegetation. Prokaryotic network exhibited a unimodal model to solar park installment while fungal network presented more resistance. The network topological features, in addition to habitat environment or microbial community structures, had significant correlations with microbial functional potentials.

## Data availability statement

The datasets presented in this study can be found in online repositories. The names of the repository/repositories and accession number(s) can be found below: The National Genomics Data Center (NGDC) with the accession number of PRJCA010425.

## Author contributions

TiW and TaW investigated plant ecological diversity and collected soil samples. YL contributed to soil characterization, statistical analysis, and data visualization. YL wrote the first draft. DS and CD improved the manuscript. All authors contributed to the article and approved the submitted version.

## Footnotes

1
http://ieg2.ou.edu/MENA

2
https://gephi.org/
