# Supplementary material for: Solar park promoted microbial nitrogen and phosphorus cycle potentials but reduced soil prokaryotic diversity and network stability in alpine desert ecosystem
Source: Front Microbiol. 2022 Sep 8;13:976335. doi: 10.3389/fmicb.2022.976335 (PMC9493309; doi:10.3389/fmicb.2022.976335)

# Supplemental Method: R script calculating robustness of microbial co-occurrence network

library(igraph)

library(microeco)

**#DON’T CHANGE THIS CHUNK**

# 1.Develop functions -----------------------------------------------------

#1.1calculation

link.random<- function(network,rm.percent) {

id.rm<-sample(1:nrow(network), round(nrow(network)*rm.percent))

net.copy=network #keep the raw network

net.copy[id.rm,]=0; net.copy[,id.rm]=0;

adj_matrix <- as.matrix(net.copy)

adj_matrix[abs(adj_matrix) != 0] <- 1

lambda <- eigen(adj_matrix, only.values = TRUE)$values

lambda <- sort(lambda, decreasing = TRUE)

lambda_sum <- 0

N = length(lambda)

for (i in 1:N) lambda_sum = lambda_sum + exp(lambda[i])

lambda_average <- log(lambda_sum/N, base = exp(1))

lambda_average

}

#1.2permutation

link.mean.random <- function(network,rm.percent,nperm=999){

t(sapply(rm.percent,function(x){

res=sapply(1:nperm,function(i){

link.random(network=network,rm.percent=x)

})

res.mean = mean(res,na.rm = T)

res.sd=sd(res,na.rm = T)

res.se=sd(res)/(nperm^0.5)

result<-c(res.mean,res.sd,res.se)

names(result) <- c("link.mean","link.sd","link.se")

result

}))

}

avgD.random <- function(network,rm.percent) {

id.rm<-sample(1:nrow(network), round(nrow(network)*rm.percent))

net.copy=network #keep the raw network

net.copy[id.rm,]=0; net.copy[,id.rm]=0;

g1 <- graph_from_adjacency_matrix(as.matrix(net.copy),weighted = T,mode = "undirected",diag = F)

average_degree1 <- mean(degree(g1))

average_degree1

degree(g1)

}

#

avgD.mean.random <- function(network,rm.percent,nperm=100){

t(sapply(rm.percent,function(x){

res=sapply(1:nperm,function(i){

avgD.random(network=network,rm.percent=x)

})

res.mean = mean(res,na.rm = T)

res.sd=sd(res,na.rm = T)

res.se=sd(res)/(nperm^0.5)

result<-c(res.mean,res.sd,res.se)

names(result) <- c("avgD.mean","avgD.sd","avgD.se")

result

}))

}

**# ONLY MODIFY THE INPUT DATA**

# ~~~~~~~~~~~~~~~~~~~~~~~~~~~~~#

netname = "GF_16s"

net = GF_16s_net

# ~~~~~~~~~~~~~~~~~~~~~~~~~~~~ #

# 2.load data

# 2.1 convert igraph to correlation matrix (recommend using this method) --------------------

net_cor <- as.matrix(

as_adjacency_matrix(

net$res_network, attr="weight",sparse = T))#convert dgCMatrix to matrix

# 2.2 Input and tidy data ---------------------------------------------------

## 2.1 input from gexf, must transfer to graph

g <- read.gexf("gf_network_cor.gexf")

graph <- gexf.to.igraph(g)

# 3. Calculation -----------------------------------------------------------

net_link_random <- link.mean.random(net_cor,seq(0,1,by = 0.01))

net_avgD_random <- avgD.mean.random(net_cor,seq(0,1,by = 0.01))

# 4.Output ----------------------------------------------------------------

net_link_avgD_random<-as.data.frame(cbind(precent=seq(0,1,by=0.01),net_link_random,net_avgD_random))

write.csv(net_link_avgD_random,paste0(netname,"_rmRandom_link_avgD.csv"))

# Supplemental Figures and Tables

Fig.S1. The relative abundance of prokaryotic (A) and fungal (B) taxonomic community structure.


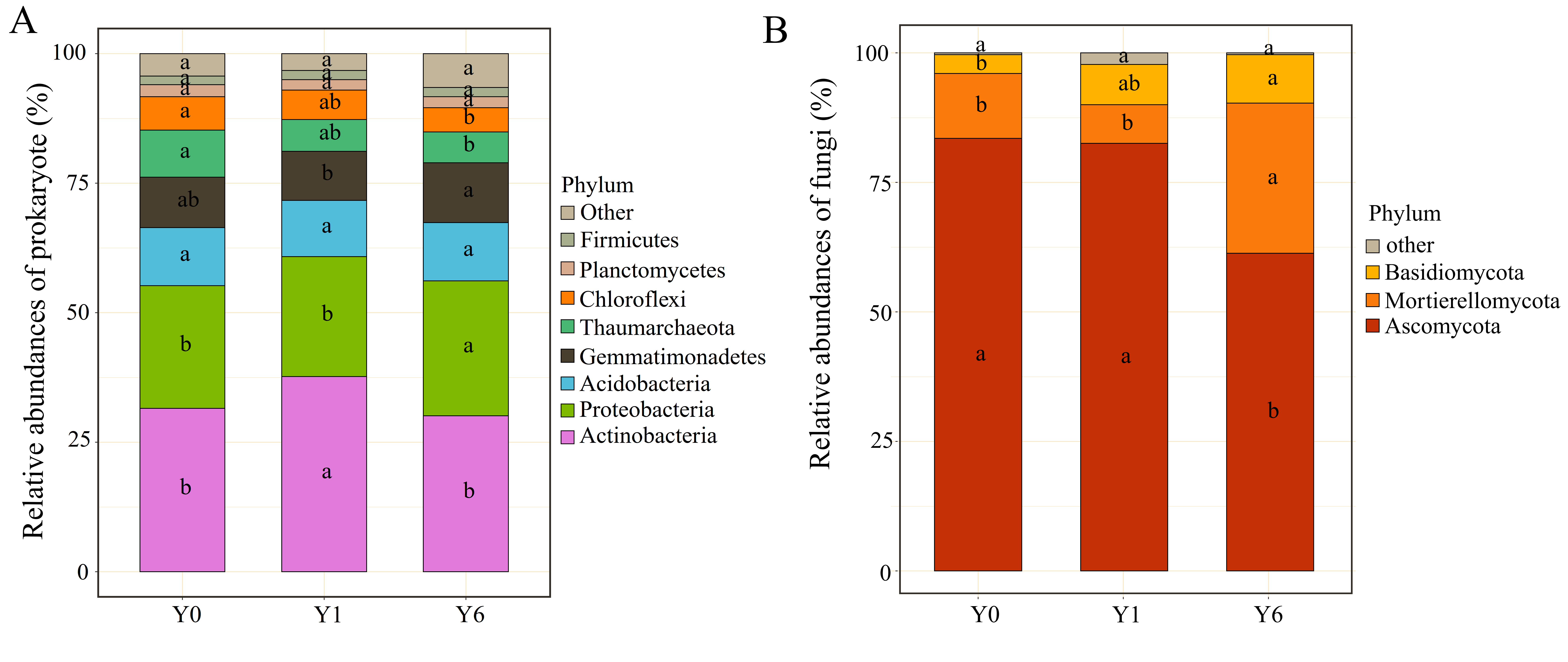


Fig.S2 RDA between prokaryotic and fungi alpha diversity index and environmental factors. (A) is the RDA between prokaryotic alpha diversity and environmental characteristics; (B) is the RDA between fungal alpha diversity and environmental characteristics. Features with suffix '.pro' and '.fun' indicate prokaryotic and fungal features, respectively.


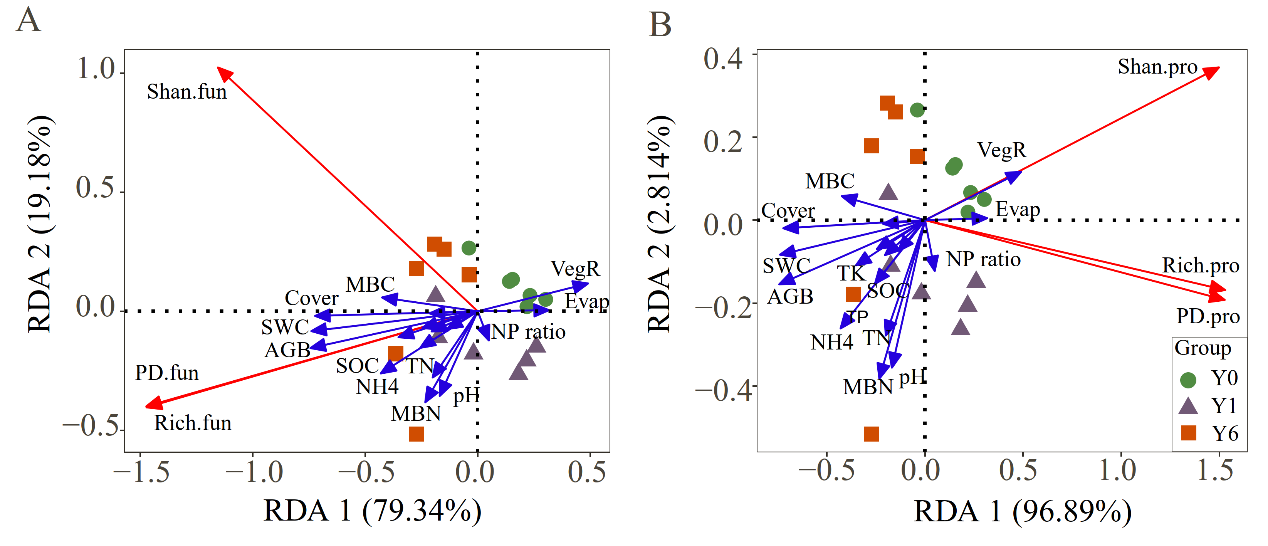


Table S1 Results of interactive-forward-selection of RDA between soil EEAs and environmental factors as well as microbial diversity

|  | Properties | Estimate | Std.Error | t value | Pr(>\|t\|) |  |
| --- | --- | --- | --- | --- | --- | --- |
| Prokaryote | (Intercept) | 0 | 0.1629 | 0 | 1 |  |
|  | SWC | -0.6527 | 0.1851 | -3.526 | 0.00336 | ** |
|  | MBN | 0.4375 | 0.1808 | 2.42 | 0.02973 | * |
|  | TP | -0.3556 | 0.173 | -2.055 | 0.05904 | . |
| Fungus | (Intercept) | 0 | 0.1454 | 0 | 1 |  |
|  | SWC | 0.3874 | 0.1919 | 2.019 | 0.06857 | . |
|  | TN | 1.376 | 0.3041 | 4.524 | 0.000866 | *** |
|  | SOC | -0.982 | 0.2708 | -3.626 | 0.003982 | ** |
|  | TP | -0.4252 | 0.1657 | -2.566 | 0.026228 | * |
|  | MBN | -0.6266 | 0.2488 | -2.518 | 0.028571 | * |
|  | NO3 | 0.4762 | 0.2251 | 2.115 | 0.058031 |  |

Table S2 Mantel Correlation between environmental properties and prokaryotic and fungi community composition based on Unweighted UniFrac distance (unwei), Weighted UniFracdistance (Wei), jaccard distance(jaccard) and bray-curtis distance (bray), respectively. Bold character indicates significant correlation at 0.05 level.

| Env | Prokaryote | | | | Fungus | | | |
| --- | --- | --- | --- | --- | --- | --- | --- | --- |
| Propoties | unwei | wei | jaccard | bray | unwei | wei | jaccard | bray |
| Full model | **0.50** | **0.35** | **0.48** | **0.41** | **0.50** | **0.35** | **0.28** | **0.47** |
| AGB | **0.50** | **0.36** | **0.48** | **0.43** | **0.48** | **0.57** | **0.30** | **0.53** |
| Cover | **0.55** | **0.36** | **0.52** | **0.41** | **0.30** | **0.37** | **0.17** | **0.36** |
| VegR | 0.10 | 0.10 | 0.10 | 0.10 | **0.28** | **0.28** | **0.26** | **0.28** |
| VegS | 0.08 | 0.11 | 0.07 | 0.07 | **0.31** | **0.27** | **0.28** | **0.31** |
| VegP | 0.05 | 0.11 | 0.04 | 0.04 | **0.30** | **0.25** | **0.28** | **0.30** |
| SOC | -0.12 | -0.01 | -0.11 | -0.06 | -0.09 | 0.01 | -0.02 | -0.07 |
| TN | -0.16 | -0.06 | -0.16 | -0.04 | 0.15 | 0.13 | 0.12 | 0.16 |
| TP | -0.06 | -0.14 | -0.07 | -0.06 | -0.16 | -0.01 | -0.19 | -0.10 |
| CN ratio | -0.10 | -0.04 | -0.11 | -0.12 | -0.08 | 0.00 | -0.03 | -0.08 |
| CP ratio | -0.04 | 0.00 | -0.04 | 0.02 | -0.13 | -0.03 | -0.06 | -0.09 |
| NP ratio | -0.08 | -0.02 | -0.08 | 0.03 | -0.13 | -0.03 | -0.16 | -0.09 |
| NH4 | 0.13 | 0.32 | 0.16 | 0.16 | 0.02 | 0.04 | 0.12 | 0.04 |
| NO3 | 0.29 | 0.03 | **0.30** | 0.15 | -0.14 | -0.03 | -0.10 | -0.09 |
| MBC | 0.18 | 0.04 | 0.15 | 0.10 | -0.03 | 0.01 | 0.08 | 0.01 |
| MBN | 0.02 | 0.15 | 0.06 | 0.07 | -0.09 | -0.03 | 0.08 | -0.05 |
| TK | 0.07 | -0.04 | 0.08 | 0.07 | -0.09 | -0.04 | -0.14 | -0.06 |
| pH | 0.03 | 0.08 | 0.07 | 0.08 | -0.12 | -0.13 | -0.20 | -0.16 |
| SWC | **0.52** | **0.36** | **0.53** | **0.48** | **0.48** | **0.61** | **0.25** | **0.54** |
| Evap | -0.12 | 0.02 | -0.12 | -0.15 | **0.26** | 0.10 | 0.13 | 0.18 |
| ST | -0.13 | -0.02 | -0.12 | -0.13 | 0.16 | **0.11** | 0.06 | 0.09 |

Figure S3 ANOVA of predicted function of soil βg enzyme


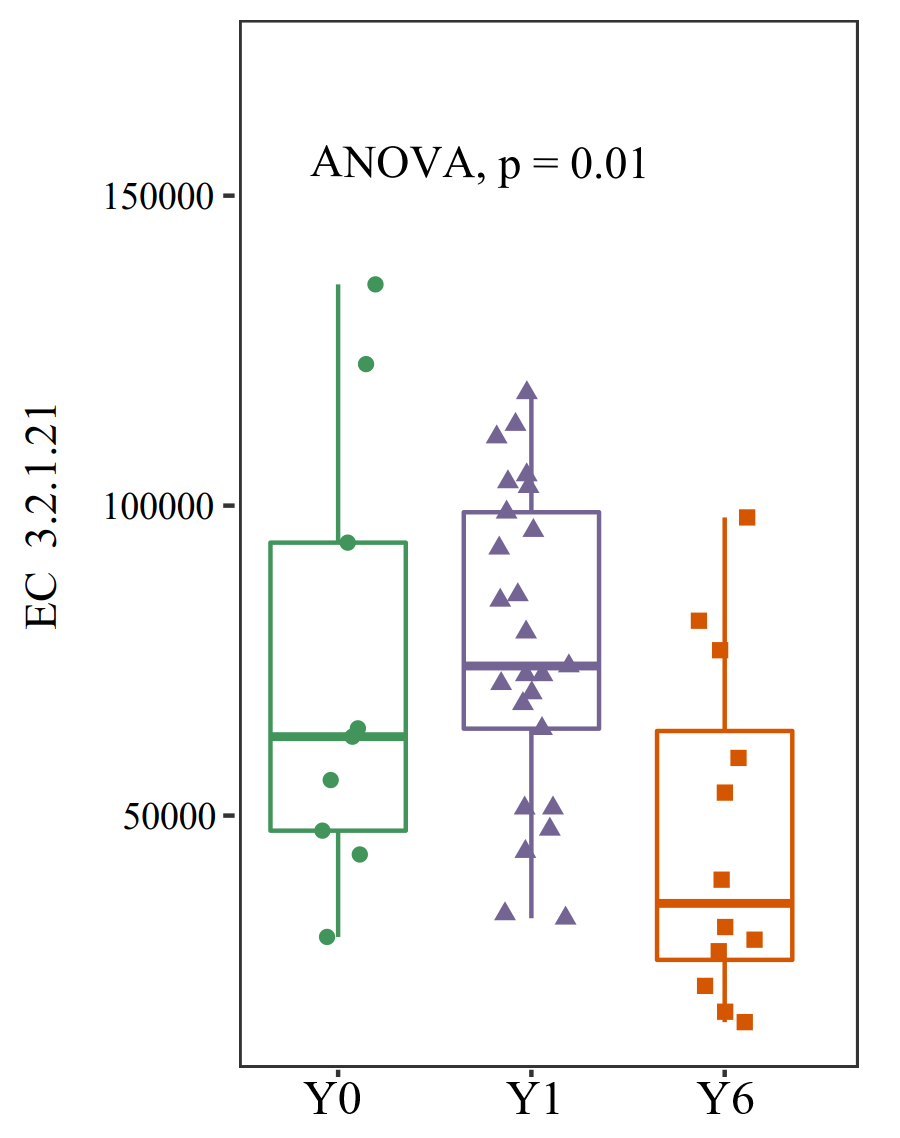


Figure S4 Mantel analysis among soil EEAs, environmental characteristics and microbial matrix. (A-C) are the mantel analysis between carbon, nitrogen, phosphorus EEAs and environmental characteristics, microbial diversity and network features, respectively.


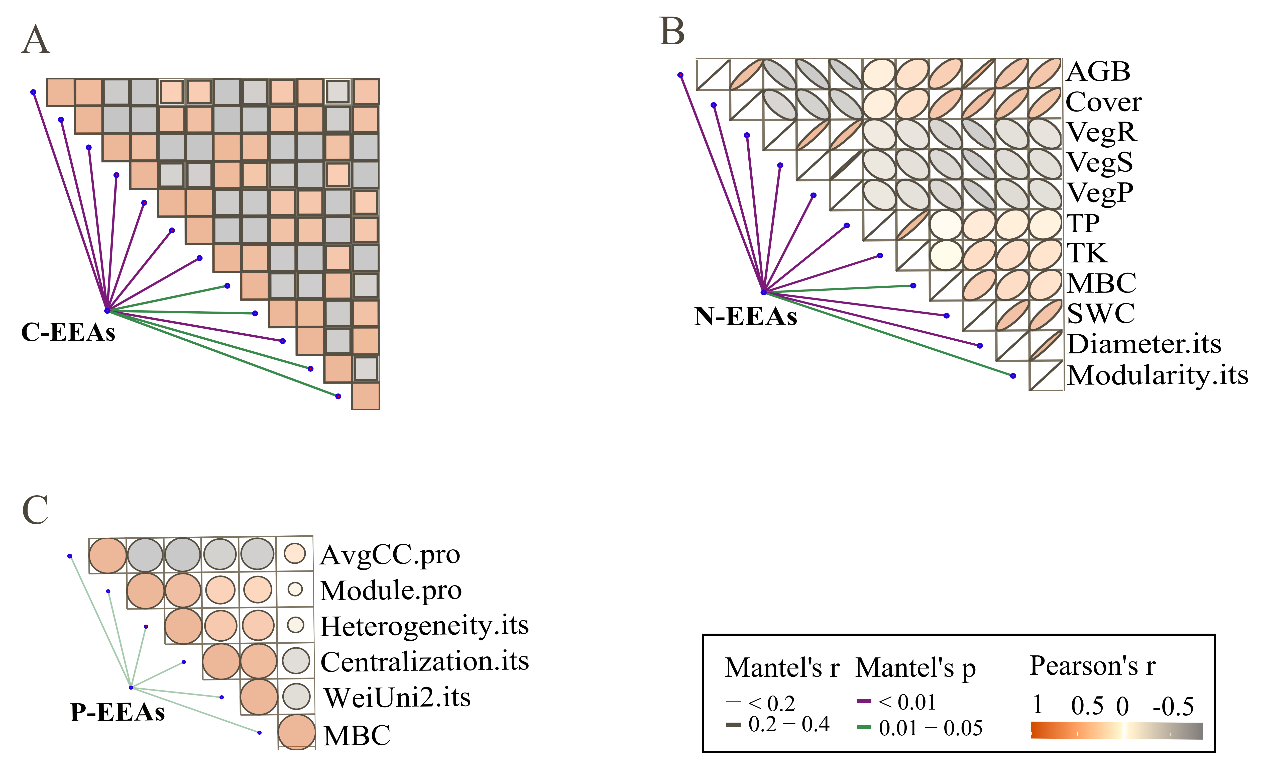


Figure S5 Canonical Correlation Analysis (CCA) between environmental factors and microbial matrix. The arrow in blue were microbial matrix, the arrow in red were environmental factors contributed most to microbial matrix. Microbial features with suffix '.pro' and '.fun' indicate prokaryotic and fungal features, respectively. MBC and MBN indicate microbial biomass carbon and nitrogen, respectively; AGB indicate plant above-ground biomass; Cover indicate plant cover; VegS indicate plant Shannon diversity index; SOC indicate soil total organic carbon; NO3 indicate soil nitrate nitrogen; NH4 indicate soil ammonium nitrogen.


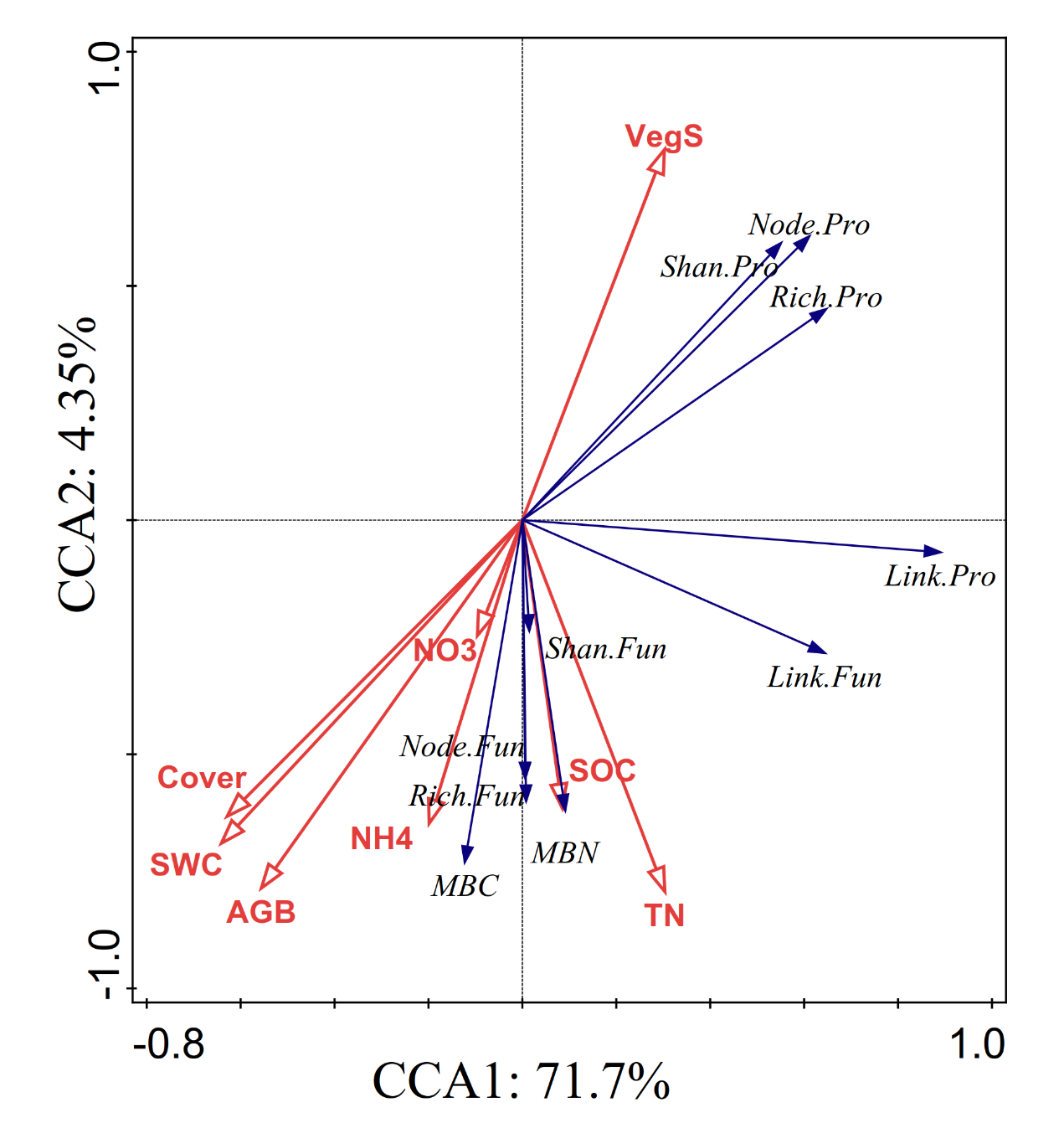


Figure S6 Heatmap of correlations between microbial phylum and soil EEAs. * indicate a significant correlation at 0.05 level.


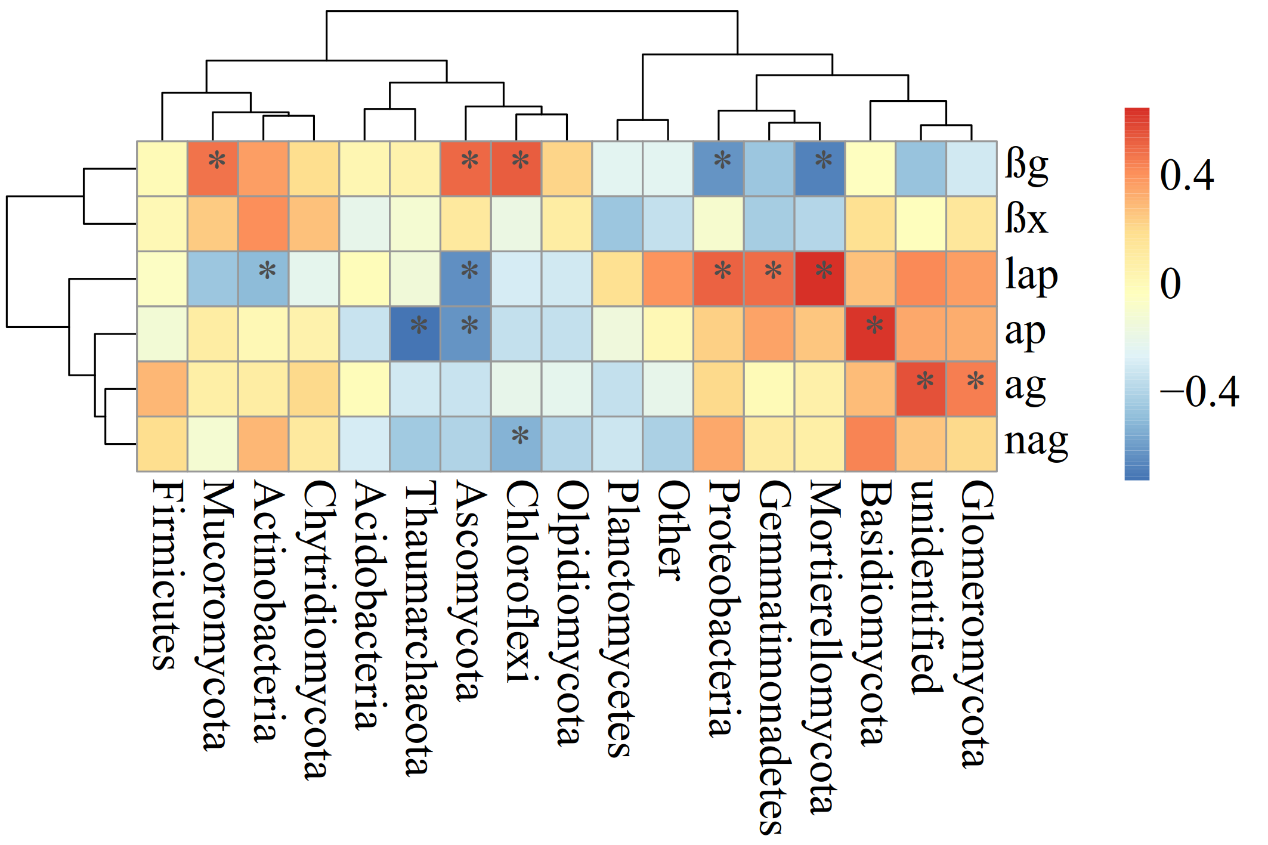

Supplement: Supplementary file 1 [file Data_Sheet_1.docx]
